# The sequential microbial breakdown of pectin is the principal incident during water retting of jute (*Corchorus* spp.) bast fibres

**DOI:** 10.1186/s12870-024-04970-4

**Published:** 2024-04-17

**Authors:** Subhojit Datta, Lipi Chattopadhyay, Shrestha Barai, Kunal Mandal, Gouranga Kar, Bijan Majumdar

**Affiliations:** 1 Biotechnology Unit, Division of Crop Improvement, ICAR – Central Research Institute for Jute and Allied Fibres, Barrackpore, 700 121 West Bengal India; 2 Division of Crop Production, ICAR – Central Research Institute for Jute and Allied Fibres, Barrackpore, 700 121 West Bengal India; 3 Division of Crop Protection, ICAR – Central Research Institute for Jute and Allied Fibres, Barrackpore, 700 121 West Bengal India; 4 ICAR – Central Research Institute for Jute and Allied Fibres, Barrackpore, 700 121 West Bengal India

**Keywords:** Bast fibre, Colonization, Interaction, Pectinolysis, Retting

## Abstract

The extraction of bast fibres such as jute from plant stems involves the removal of pectin, hemicellulose, and other noncellulosic materials through a complex microbial community. A consortium of pectinolytic bacterial strains has been developed and commercialized to reduce the retting time and enhance fibre quality. However, there are currently no studies on jute that describe the structural changes and sequential microbial colonization and pectin loss that occur during microbe-assisted water retting. This study investigated the stages of microbial colonization, microbial interactions, and sequential degradation of pectic substances from jute bark under controlled and conventional water retting. The primary occurrence during water retting of bast fibres is the bacterially induced sequential breakdown of pectin surrounding the fibre bundles. The study also revealed that the pectin content of the jute stem significantly decreases during the retting process. These findings provide a strong foundation for improving microbial strains for improved pectinolysis with immense industrial significance, leading to a sustainable jute-based “green” economy.

## Introduction

Pectin is not only a vital component of cell walls that provides strength to plant stems; emerging evidence suggests that pectin and other cementing materials are critical for determining the yield and quality of plant stem fibres [1, 2]. Controlled disassembly of pectin from stem cells is crucial for the separation of bast fibres from the woody core. Jute (*Corchorus olitorius*, *C. capsularis*) fibre is the world’s most significant source of lignocellulosic bast fibre; it is produced from plant bark and is completely biodegradable, making it a green material of choice. In Southeast Asia, particularly in India and Bangladesh, jute cultivation considerably improves the socioeconomic well-being of millions of agricultural households, and it is the second most valuable naturally occurring fibre after cotton [3]. Through a microbial pectinolysis and decohesion process called retting, fibre separation is achieved at a low cost in a hydrated environment [4]. Due to the combined action of water and aquatic microbes, fibres are separated from adjacent nonfibrous tissues by removing pectins, hemicelluloses, gums, and mucilaginous substances [3, 5]. In the case of the bast fibre of flax, different pectin compounds in the parenchyma around the fibre bundles and the cambium decompose during retting, and their quantity decreases from 3 to 4% to 0.5-1% of stem dry matter [6]. However, to date, there has been no such systematic study on pectin degradation during water retting in jute.

Microbe-mediated retting of bast fibres can be achieved by either water retting or dew retting methods. In central-northern European countries, the dew-retting of flax straw is achieved by spreading the straw on the ground, and pectins are broken down by pectinolytic microorganisms, mainly filamentous fungi [7]. However, in the Gangetic Plain of India and Bangladesh, the separation of jute fibres is almost invariably achieved in water bodies in the presence of naturally occurring aquatic pectinolytic microorganisms. The breakdown of pectin glued around the fibre bundles is the principal incident of retting. There are few reports on the sequential breakdown of pectin leading to the separation of fibres from the stems of bast fibre plants, including jute, during microbial water retting and associated structural changes. A combination of microbes secreting a variety of enzymes is more efficient for the retting of bast fibre plants than a single species of bacteria or fungus [8]. The degradation of pectic substances during retting was previously used to define the degree of retting, and subsequent scanning electron microscopic studies structurally revealed the degradation of pectic substances during water retting of flax [9]. However, a more detailed analysis is needed to understand the microbial interactions and the sequential morphological changes that occur in the bark during water retting of jute plants. Here, we present a detailed analysis to help understand the microbial interactions and sequential morphological changes that occur in the bark during water retting of jute plants.

We previously developed an effective microbial retting consortium, commercially called ‘CRIJAF SONA’, to efficiently produce high-quality fibre in a short duration. A microbial consortium consisting of three novel pectinolytic bacterial strains was isolated from jute retting water; this microbial consortium has very high levels of polygalacturonase (PG), pectin lyase (PNL) and xylanase activities without any cellulase activity and was found to be very useful for retting jute and mesta [10]. Retting with CRIJAF SONA has been demonstrated under farmers’ field conditions across the jute and mesta growing states of India and found to be very useful for quick retting with quality fibre production [11, 12]. The genome sequencing of the constituent bacteria (three species of *Bacillus*) revealed that the genome sizes of those bacterial strains are ∼ 3.8 Mb, with 3729 to 4002 protein-coding genes. Genome sequencing also confirmed that retting bacteria degrade pectin, hemicellulose and other noncellulosic materials and thus are not harmful to fibre production [13]. In large-scale jute-retting trials with and without microbial retting consortia, the sequential changes in enzymatic activity indicated increased PNL (123.1 IU/ml/min ^1^), PG (3.56 IU/ml/min) and xylanase (0.818 IU/ml/min) activities during the middle stage of retting (5–7 days) and very low activities at the last stage of retting (11–14 days). The PG, PNL and xylanase enzymes released by the microbial consortium during the retting of jute helped in the faster biodegradation of pectin and xylan than did those released during the control retting [14].

Notwithstanding the popularity of this consortium, it is well recognized that microbial strains need further improvement for quicker retting and finer fibre production. The current study provides a better understanding of the structural changes in a jute stem that occur during water retting due to the breakdown of pectin caused by the activities of aquatic microbes. In this study, the jute stems were retted in the laboratory under controlled conditions. Bark samples were taken on day 0 (pre-retting), day 7 (mid-retting), and day 10 (end-retting) of the retting period as reported earlier [14], and subsequent analysis of the samples was performed *via* light and scanning electron microscopy (SEM). Longitudinal and cross-sectioned jute bark samples were studied to determine the stages of microbial colonization, microbial interactions and sequential degradation of pectic substances. The pectin content of non-retted bark and retted fibre samples was estimated. The activity of pectinolytic and xylanolytic enzymes secreted in the retting water and the monosaccharide content in the retting water were also quantified.

The gradual microbial colonization, microbial interaction, and breakdown of pectic materials surrounding and between the fibre bundles during the retting phase are all thoroughly described in this study. Bast fibre decohesion from the epidermis’s core does not begin until pectin molecules are cleared from around and between fibre bundles. The scenario of retting as well as the structural changes in the bark to fibres that take place during the retting process by the action of bacteria are elaborately depicted. These results may be useful in determining the ideal retting time while avoiding overretting to create stronger, finer, and better-quality jute fibres.

## Materials and methods

### Plant material, retting method and sampling

Dark jute (*Chorchorus olitorius* L., cv. JRO 204) was grown at the ICAR - Central Research Institute for Jute and Allied Fibres, Barrackpore, India (22.7745° N, 88.5148°E) from April to August, and mature plants (120 days old) were harvested. According to the IUCN Policy Statement and the Convention on the Trade in Endangered Species, the studied species, *Chorchorus olitorius*, is not classified as endangered or at risk of extinction, and the study complies with relevant institutional, national, and international guidelines and legislation. One kilogram of 10–12 cm long jute stem piece was immersed in 10 L of water in a tray, and controlled retting was performed in an incubator at 34 °C. To reduce the retting period, a previously commercialized microbial consortium (CRIJAF SONA) composed of *Bacillus safensis*, *Bacillus velezensis* and *Bacillus altitudinis* was used. For 1 kg of jute stem, 400 µl of spore suspension (10^9^ spores/ml) of the above three strains was used at a 1:2:1 ratio. For comparative studies on microbial colonization progression and interactions, samples were also collected from naturally occurring retting ponds without the bacterial consortium. Bark samples were taken to assess microbial colonization, anatomical changes and pectin degradation on days 0, 7, and 10 (or at the end of the retting period for microbial colonization during conventional retting) of the retting period. All the experiments were carried out in triplicate.

### Pectin content of jute bark samples

The method of [15] was slightly modified to extract and measure the pectin content from jute bark samples at different stages of retting. Stems and retted fibre samples were collected from the retting trays. The bark was separated from the woody core, blotted, and oven-dried at 50 °C overnight. Dry samples (0.6 g) were chopped into pieces and extracted three times with 80% alcohol at 75 °C for one hour each. The mixture was allowed to cool to room temperature before being centrifuged for 15 min at 8000×g. The supernatant was discarded, and the pellet (alcohol insoluble residue) was suspended in 20 ml of double distilled water, incubated at 50 °C for 30 min, and then centrifuged at 8000xg for 15 min. The supernatant was transferred into a 100 ml conical flask and served as the source for calculating the amount of water-soluble pectin. The insoluble fraction was dried overnight at 50 °C and extracted for an hour at 100 °C with 5% ammonium oxalate. Following saponification in 5 N NaOH, at room temperature, 200 µl of the saponified extract was mixed with 3.0 ml of H_2_SO4, and 100 µl of carbazole reagent was added to make up the final reaction mixture. For soluble pectin, 200 µl of supernatant was used. After heating to 60 °C, the reaction mixture was immediately cooled under running water. The absorbance at 535 nm was recorded using a GENESYS™ 180 UV‒Vis Spectrophotometer (Thermo Fisher, MA, USA). A standard curve was drawn using galacturonic acid as the reference.

### Activity assays of pectin- and hemicellulose-degrading enzymes

The activity of pectinolytic and xylanolytic enzymes secreted in the retting water was assessed by using standard protocols [16]. The retting water samples were drawn from the retting trays on days 0, 7, and 10 of the retting phase and centrifuged at 8000×g for 5 min to obtain the cell-free supernatant as an enzyme source.

### Polygalacturonase assay

Polygalacturonase activity was determined in the retting liquor by mixing 250 µl of cell-free supernatant with 500 µl of 1% polygalacturonic acid sodium salt (Sigma‒Aldrich, USA; methyl esterification value 15%) dissolved in 10 mM Tris–HCl buffer (pH 8.0) and 250 µl of the same buffer. The concentration of reducing sugars in the solution was determined spectrophotometrically [10, 16]. As an enzyme blank, heat-inactivated supernatant (100 °C for 30 min) was used. One unit (IU) of polygalacturonase activity corresponds to the release of 1 µmol of galacturonic acid/min/ml of retting liquor. The calibration reference was D-galacturonic acid (Sigma‒Aldrich, U.S.A.).

### Xylanase assay

Xylanase activity was measured by mixing the cell-free supernatant with an equal volume of beech-wood xylan (1% w/v; Sigma Aldrich, U.S.A.) dissolved in 0.1 M phosphate buffer (pH 6) and incubating at 50 °C for 10 min in a water bath. The test tubes were then placed in boiling water for another 10 min, after which the reducing sugar content was estimated via the DNS method [16]. The amount of enzyme that released one µmol of D-xylose per minute during the enzyme-substrate reaction was defined as one unit of xylanase activity (IU/ml/min). D-xylose (Himedia, Mumbai, India) was used as a standard reference [17].

### Reducing sugar concentration

Retting water samples were collected on days 0, 7 and 10, and the reducing sugar concentration in the retting liquor was determined via the DNS method [16].D-galacturonic acid (Sigma‒Aldrich) was used as the standard.

### Bacterial concentration in the retting water

The retting water microbial load, enumerated as colony forming units (CFU), was standardized earlier [3]. The culturable pectin- and xylan-degrading bacteria and the total culturable microbial population were counted throughout the experiment. On day 0, day 7 and day 10, the retting water samples were collected, diluted in sterile distilled water and spread on pectin agar, xylan agar and nutrient agar plates. Colonies were counted after 48 h of incubation at 34 °C in a colony counter using standard plate count method [3]. To ensure accuracy and reproducibility of results, work area was properly sterilized, appropriate dilution was adopted, and the colony counter was calibrated with known bacterial samples.

### Scanning electron microscopy of jute bark samples

Plant tissue samples were collected at a specified time after retting, immediately fixed in formaldehyde, alcohol, and acetic acid (FAA) and stored in 70% ethanol until use. Longitudinal and transverse sections were made with a razor blade, and critical-point drying was performed. The dried samples were adhered to stubs with the help of sticky tape, coated with gold particles and viewed under a scanning electron microscope (Zeiss Gemini SEM 450 and Gemini 2, Carl Zeiss AG) [18].

### Histological staining of jute bark samples

The bark samples were embedded in paraffin wax (Merck, Mumbai, India), and sectioning was performed using a rotary microtome (RM2135, Leica Biosystems). To locate the areas of pectin degradation, sections were stained with an aqueous solution of ruthenium red (0.05%) and counterstained with toluidine blue O (0.02%) [15]. The stained tissues were mounted on clean glass slides with a drop of glycerol. The stained sections of the jute bark were observed under a light microscope (BX43, Olympus Life Sciences) and photographed.

## Results

**Fig. 1:**
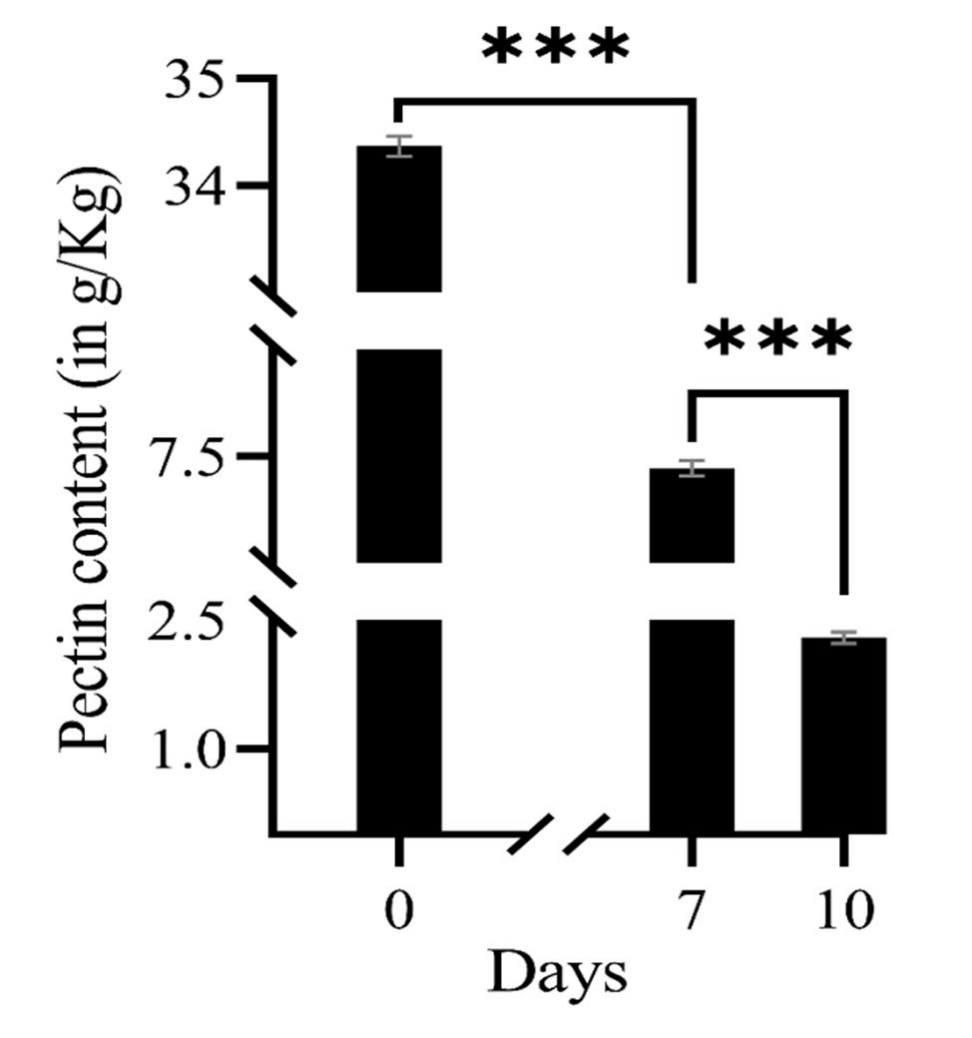
Pectin content of jute bark samples during retting. After seven days of retting, the pectin content of the bark samples decreased substantially. GraphPad Prism 8 was used for data analysis and graph design

Under laboratory conditions, the retting of jute stems was completed after 10 days using a microbial consortium (CRIAF SONA). Bark samples collected after 0, 7, and 10 days of incubation presented different stages of retting. The initial pectin content of the jute bark, which was 34.4 mg/g on day 0 of retting, decreased to 7.4 mg/g after day 7 and to 2.3 mg/g after completion of the retting process (Fig. 1). This reduction accounted for almost 78% and 93% of the pectin degradation on day 7 and day 10 of retting, respectively. Only 0.1% of the dry weight of non-retted bark (day 0) was made up of the water-soluble fraction obtained from AIR pectin. However, this fraction was not detectable in samples from later stages of retting (Table 1).

### Enzyme assays

**Fig. 2:**
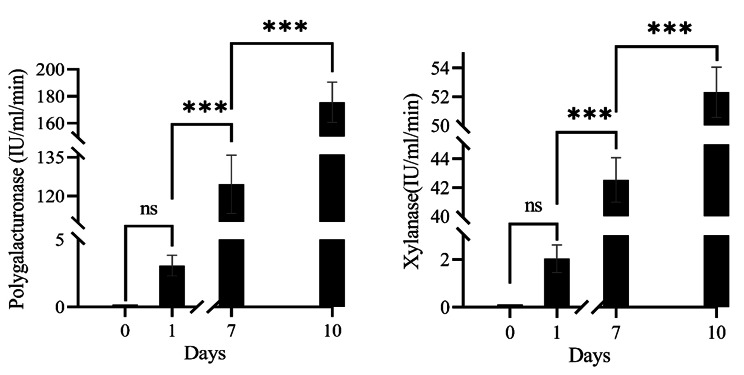
Enzyme activities in the retting water during jute retting. (**a**) Polygalacturonase activity during retting; (**b**) Xylanase activity during retting. As the retting process progresses, the activity of the enzymes in the retting liquor increases gradually. GraphPad Prism 8 was used for the data analysis and graph preparation

The activity of polygalacturonase and xylanase in the retting water was measured during the retting experiment (Fig. 2). The initial activities of polygalacturonase and xylanase were 3.08 IU/ml/min and 2.02 IU/ml/min, respectively. As the retting process progresses, the activity of these enzymes in the retting liquor increases gradually. On the 7th day of retting, the polygalacturonase activity was 124.47 IU/ml/min, the xylanase activity was 42.53 IU/ml/min, and finally, the activity reached 175.72 IU/ml/min and 52.32 IU/ml/min on the 10th day of the retting period, respectively.

### Concentration of reducing sugars and bacterial count in the retting water

The amount of sugar released during retting is an indicator of microbial depolymerizing activities in the retting water. As retting progressed, the bacterial count in the retting water increased steadily (Table 2). The concentrations of both pectin and xylan degraders in the retting water peaked on the 10th day of incubation (12.11 and 9.13 log cfu ml^-1^, respectively). A concomitant increase in reducing sugars in the retting water was also observed during this period. The high correlation coefficients between the pectin- and xylan-degrader concentrations and reducing sugar content in the reting water (0.93 and 0.94, respectively) indicated that these factors were linearly related.

### Scanning electron microscopy of jute bark and fibre

#### Microbial colonization

**Fig. 3:**
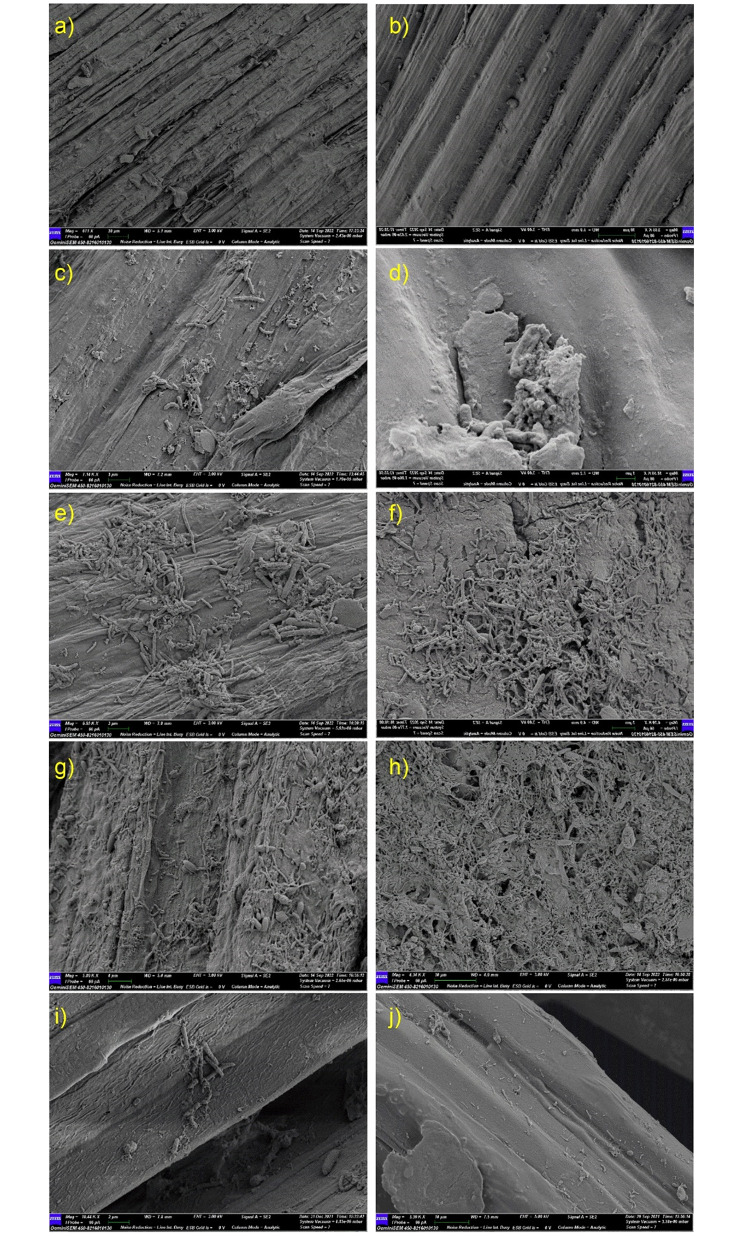
SEM micrograph of microbial colonization (conventional retting) on jute bark. (**a**, **c**, **e**, **g**, **i**) Inner surfaces of jute bark; (**b**, **d**, **f**, **h**, **j**) Outer surfaces of jute bark; (**a**, **b**) Inner and outer surfaces of fresh jute bark showing fibre boundaries with intact cementing material around them; no microbiological growth was observed. (**c**, **d**) Inner and outer surfaces of jute bark on day 3 of the retting period; scattered microbial structures were found on both surfaces. (**e**, **f**) Inner and outer surfaces of jute bark on day 5 of the retting period. Minor cracks were found on the outer surface, and microbial growth occurred on both sides of the jute stems. (**g**, **h**) Inner and outer surfaces of jute bark on day 7 of the retting period. Microbial mats were visible on the outer side of the stems, and bacterial cells and fungal spores were observed adhering to the inner surface. The outside surface was found to have perforations. (**i**, **j**) Inner and outer surfaces of the retted jute fibre. The retting process took 18 days to complete in the case of conventional retting without microbial formulation

At the beginning of the retting process (day 0), longitudinal sections of fresh jute bark samples (conventional retting method) showed intact cellular structures in the SEM micrographs (Fig. 3). No microbial colonies were discerned on the outer or inner surfaces. Early in the retting process (day 3), scattered microbial structures were found on the inner surface of the jute bark, whereas bacterial colonization along with fungal hyphae was observed on the outside surfaces. Deformity on the outer surface of the bark occurred during this period, suggesting partial degradation of cellular components. SEM micrographs of mid-retted (day 5-day 7) bark samples revealed the establishment of microbial colonies on the inner surfaces and distorted exterior surfaces covered by microbial mats and fungal spores. Micrographs of the completely retted (day 18) fibre samples showed that the fibre bundles dissociated from one another, and random microbial colonies were found on both surfaces.

**Fig. 4:**
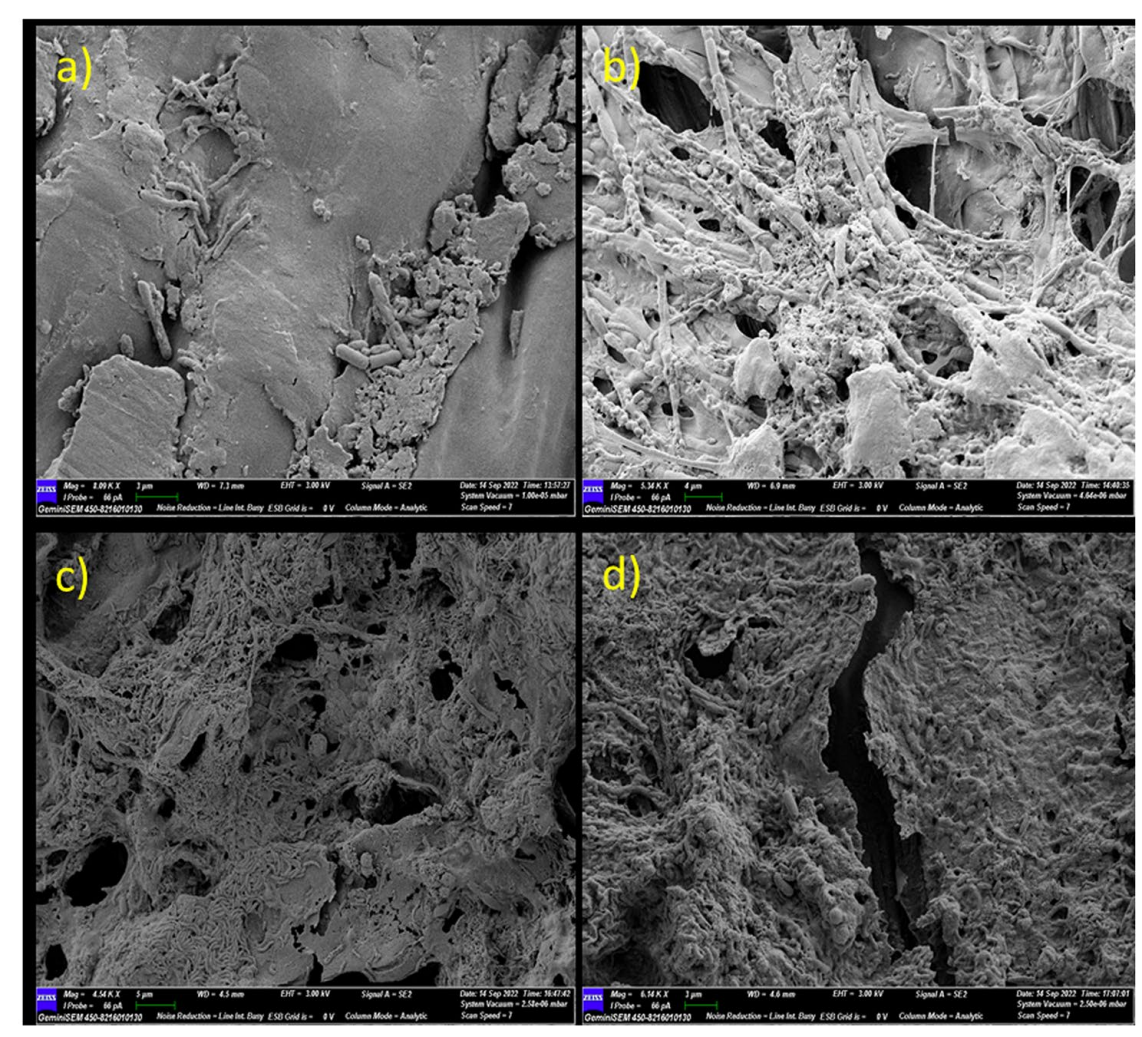
SEM micrographs of microbial colonization progression and microbial interactions in comparative studies (between conventional retting and retting with microbial formulation). (**a**) Outer surface of jute bark on day 3 (conventional retting method without microbial formulation); on the surface of the jute stem, fewer microbial cells were observed. (**b**) Outer surface of jute bark on day 3 (retting with microbial formulation); dense mycelial growth and bacterial colonization were both observed. (**c**) Outer surface of jute bark on day 7 (conventional retting method without microbial formulation). (**d**) Outer surface of jute bark on day 7 (retted with microbial formulation). In contrast to conventional retting, retting with microbial formulation results in a substantially faster rate of colonization on bark samples and cell wall degradation. After day 7, there was no significant difference in microbial colonization on either surface between the two groups of patients

In the early stages of water retting with microbial formulation, significant variations were discovered. Figure 4 depicts the bacterial colonization and mycelial growth on the outer surface of the jute barks that were retted using the microbial formulation, in contrast to the SEM images of the jute stems that were retted using the traditional retting technique, which showed less bacterial structure on the outer surface. Significant alterations were found on the outer surface of the jute bark on the 7th day of retting with the microbial formulation. Cementing materials begin to deteriorate where bacterial clusters are present. However, the removal of stems by the conventional method results in microbial colonization and perforation on the outer surface. Regarding microbial colonization, no observable difference was observed on either surface after day 7 of retting.

#### Fibre ultrastructure

**Fig. 5:**
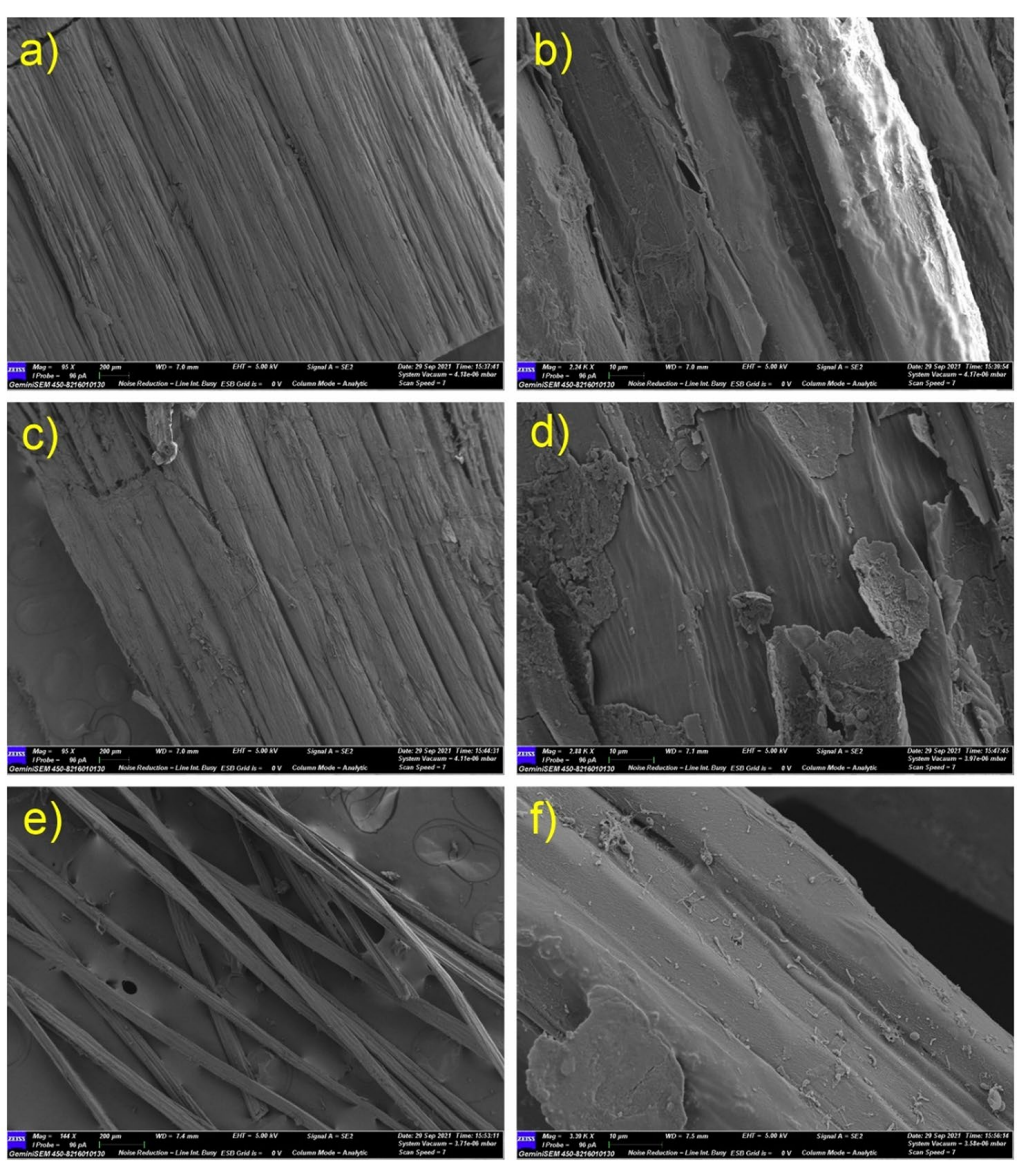
SEM micrographs (controlled retting with microbial formulation) of longitudinal sections of jute stems. (**a**, **b**) Intact epidermal layer on the outer surface of nonretted stems (day 0); (**c**, **d**) In midretted (day 7) stems, cementing material peels away from the outer surface, and fibre strands become visible; (**e**, **f**) Distinct fibre strands with no cementing material present on the outer surface in retted fibre samples (day 10)

**Fig. 6:**
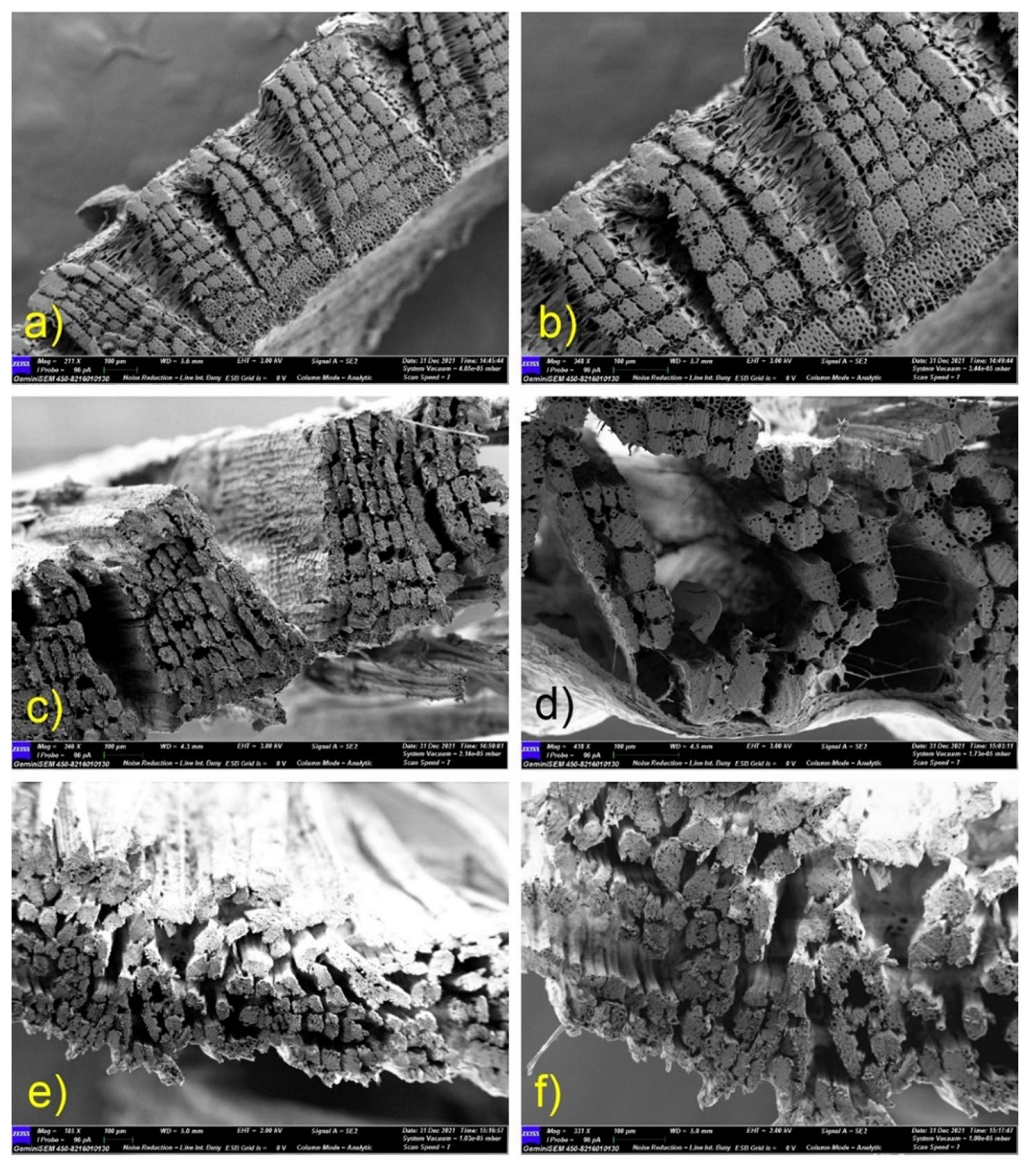
SEM micrographs of transverse sections (controlled retting with microbial formulation) of jute stems. (**a**, **b**) Nonretted stems (day 0), undamaged cementing material between and around fibre bindles; (**c**, **d**) Mid-retted (day 7) stems, absence of cementing material around fibre bindles, partial separation of fibre bindles; (**e**, **f**) Retted stems (day 10), complete separation of fibre bundles, no presence of cementing material between or around the fibre bundles

SEM images of longitudinal and transverse slices of jute bark and fibre samples were taken during the retting process to illustrate the progressive removal of the central lamella and the separation of the fibre bundles from the core and one another. Bast fibres and cementing material are intact in samples collected before retting (0-day samples), and SEM investigations reveal no microbial growth. Microbial colonization and decementing surrounding the fibre bundles are visible in longitudinal section micrographs of mid-retted samples (from day 3 to day 7) (Fig. 5). Transverse section micrographs demonstrate the partial deterioration of the middle lamella when the fibre bindles stay attached. In the retted samples (day 10), there was no cementing material present between or around the fibre bundles (Fig. 6). Extensive degradation by microbes leads to destruction of the cuticle layer.

**Fig. 7:**
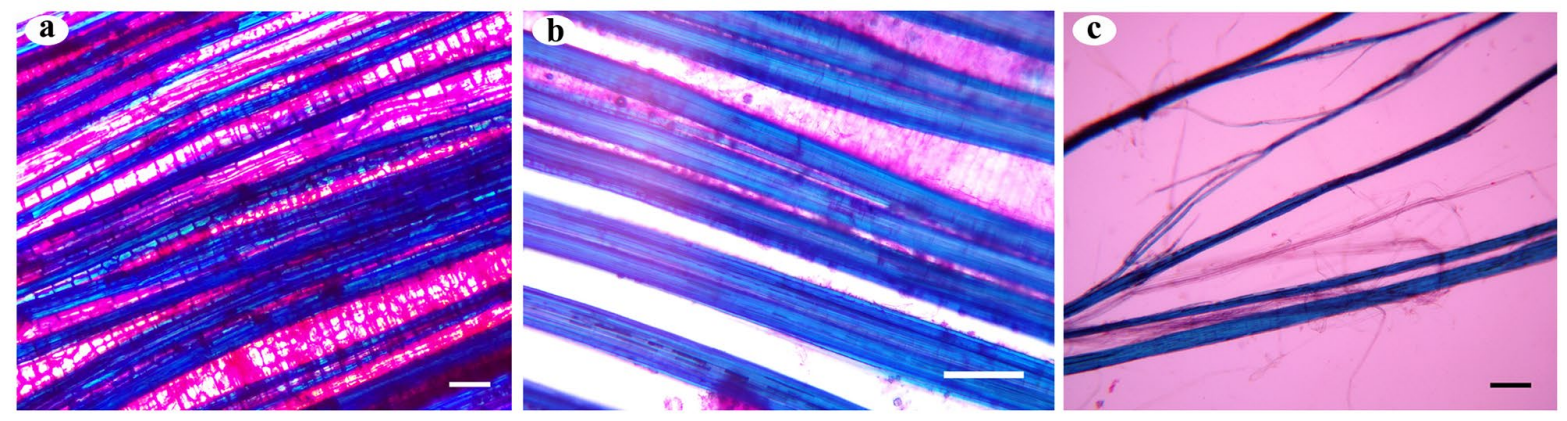
Light microscopy of longitudinal sections of jute stems. Differential staining was performed with ruthenium red and toluidine blue ‘o’. Pectin-rich parenchyma catches the color of ruthenium red, and the presence of lignin causes the fibre bundles to take blue tint of toluidine blue O: **a**) fresh stem (day 0) with intact orientation of plant tissues; **b**) mid-retted (day 7) bark samples showing gaps between the bast fibre bundles; and **c**) dissociated fibre bindles of retted fibre samples (day 10). Scale bar = 200 μm

**Fig. 8:**
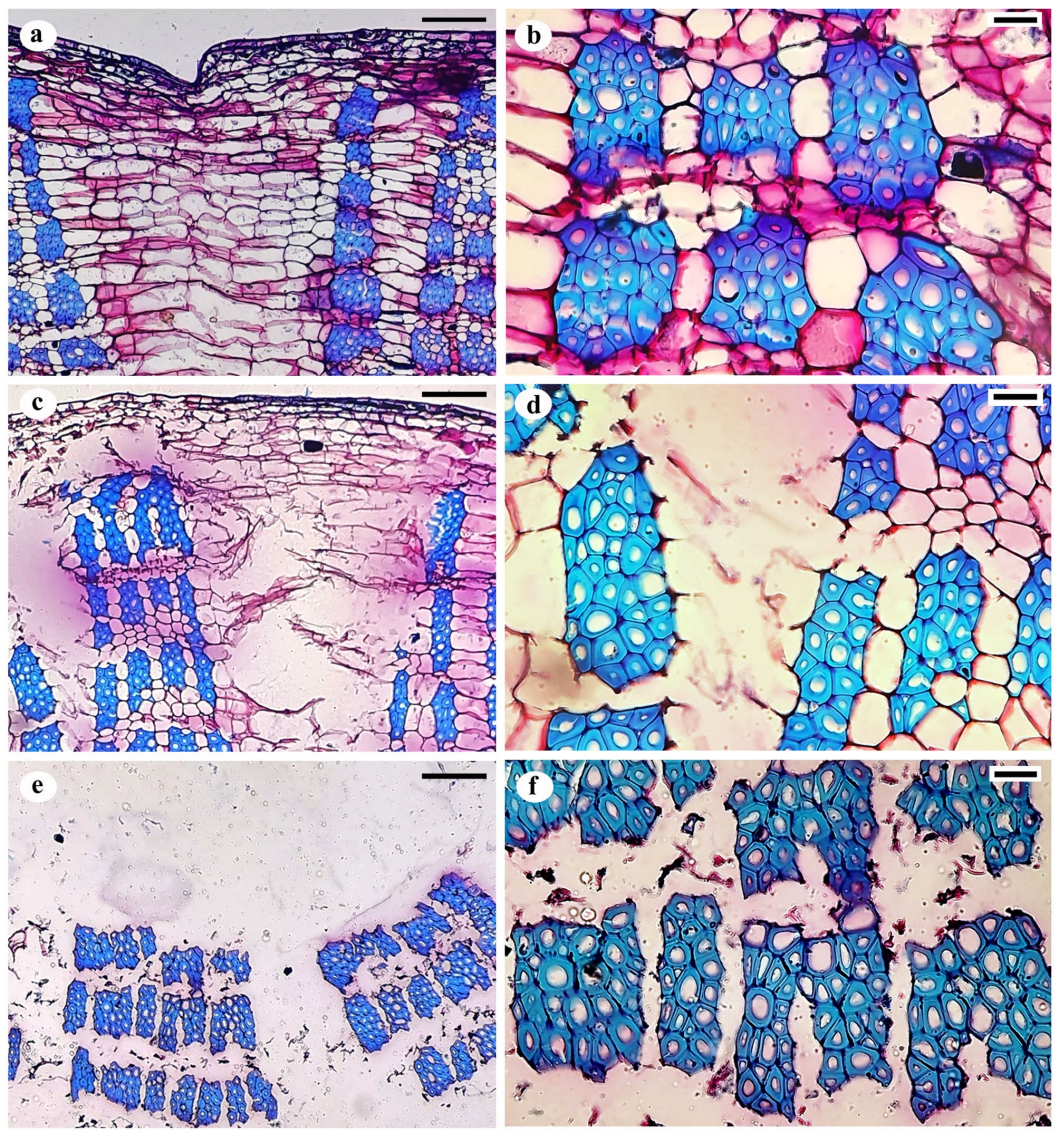
Light microscopy of transverse sections of jute stems. Differential staining was performed with ruthenium red and toluidine blue ‘o’. (**a**, **b**) Nonretted stems, intact cuticle and epidermis layer on day 0; (**c**, **d**) midretted stems with distorted epidermis and parenchyma on day 7; (**e**, **f**) Retted fibre samples without any cementing material on day 10. Scale bar = 100 μm (**a**, **c**, **e**) and 20 μm (**b**, **d**, **f**)

**Table 1:** Pectin content of jute bark and fibre

Retting duration (Days)	Pectin content of jute bark and fibre		
	Total pectin (g/kg)	Water soluble pectin(g/kg)	Percentage of reduction
Day 0	34.37 ± 0.094	1.08 ± 0.065	
Day 7	7.39 ± 0.069	**-**	78.48%
Day 10	2.3 ± 0.064	**-**	93.31%

**Table 2:** Log value of colony-forming units and reducing sugar content of the retting water

Duration (Days)	Pecting degrading bacteria(Log value of CFU/ml)	Xylan degrading bacteria (Log value of CFU/ml)	Total microbial count (Log value of CFU/ml)	Reducing sugar concentration (mg/ml)
**Day 0**	1.84 ± 0.26	1.53 ± 0.17	2.09 ± 0.04	**-**
**Day 7**	9.90 ± 0.70	7.49 ± 0.15	13.72 ± 0.17	0.168 ± 0.015
**Day 10**	12.11 ± 0.64	9.53 ± 0.18	15.26 ± 0.26	0.376 ± 0.005

## Discussion

### Light microscopy of jute bark and fibre

Differentially stained longitudinal and transverse sections of the retted fibres (0, 7th and 10th day samples) revealed anatomical changes during the retting process (Figs. 7 and 8). The lignified fiber bundles absorbed the blue hue of toluidine blue O, while the parenchyma’s cell wall pectin caused it to exhibit the color of ruthenium red.Pre-retting bark samples showed that the epidermal and cortical cells were intact, maintaining perfect integrity. The cuticle layer outside the epidermis also remained unchanged. However, gaps were observed between the bast fibre bundles and the residual epidermis in the mid-retted bark samples as the retting process progressed. The epidermis and the cortex appeared distorted due to the presence of partially damaged parenchyma in these two layers. The cuticle was less affected. The fibre bundles remained intact and, as a result, dissociated from the bark tissues. Retting revealed the absence of parenchyma cells. The cortical layers lost their integrity and were washed away, freeing the fibre bundles from one another.

This study aimed to unravel the sequential structural changes in bast tissues and progression of bacterial colonization during retting of jute in water containing specific pectinolytic bacterial strains. The success of retting is dependent on the enzymatic breakdown of cell wall pectin, which also affects the fiber’s ultimate quality. Proper retting ensures that the fibers are adequately separated without excessive damage or weakening. The basal portion of the fiber, known as “cuttings,” remains hard in the event of incorrect retting. The quality of the fiber decreases as the cutting percentage increases. Over-retting can lead to fiber breakage and reduced strength, while under-retting may leave residual pectin, resulting in fibers that are coarse and less flexible. Over degradation of pectin also affects the uniformity and texture of the jute fiber. Well-retted fibers tend to be more uniform in diameter and texture, with a softer feel and smoother surface and thus enhancing the overall appearance and value of the final jute products. The findings showed that as the retting process moves forward, there are significant changes in the way that bacteria colonize. Bast fibres are cellulosic fibres that are extracted from the phloem of fibrous plants. Several single fibres are bundled together to form a fibre bundle. The middle lamella, which acts as glue and contains pectin and lignin components, holds the fibre bundles together. The purpose of the retting procedure is to eliminate these cementing substances and separate the fibres from the woody core [2].

Pectic compounds are synthesized in the Golgi apparatus from UDP-D-galacturonic acid in young growing cell walls during the early phases of development [19], where they act as glues, facilitating cell adhesion and separation and regulating cell growth and its formation [20]. The pectin molecule is made up of a linear polymer, homogalacturonan (HG; α-1,4-linked galacturonic acid), which is the most prevalent pectic polysaccharide in nature. HG is partly methyl esterified at the O-6 position and acetyl esterified at the O-2 or O-3 position. The other pectic polysaccharides present in this molecule are significantly more complex in structure than those in HG. These included rhamnogalacturonan II (RG-II), xylogalacturonan (XGA), and apiogalacturonan (AP), as well as the structurally more variable rhamnogalacturonan I (RG-I) [21].

Pectinases are the enzymes that are mainly responsible for the degradation of pectin present in plant cell walls and can be classified through their ability to bind to the structure of pectin molecules. RG hydrolase, RG rhamnohydrolase, and RG galactouronohydrolase are active in the ‘hairy region’ of the molecule and contain rhamnogalturonan (RG) residues. Pectin methyl esterases, pectin acetyl esterases, polygalacturonases, pectin lyases, and pectate lyases aggressively breakdown the molecule’s ‘smooth region’, which contains Homogalacturonan (HG) residues. Other auxiliary enzymes involved in the breakdown of pectin side chains include α-arabinofuranosidase, endoarabinase, β**-**galactosidase, endogalactanase, feruloyl and p-coumaroyl esterases, and feruloyl and p-coumaroyl esterases [22]. The characterization of pectinases from diverse bacteria, primarily enzymes specific to smooth pectin areas, has been the subject of numerous investigations. However, very few studies on hairy pectin region-specific enzymes have been published. The most challenging aspect of developing studies with RG-degrading enzymes might have been obtaining the right substrate [23].

### Removal of pectin is the key to the retting process

Pectin, an acidic polysaccharide found in the cell wall of plants, contains both soluble and insoluble components. The soluble components foster microbial proliferation and aid in the establishment of the microbial community necessary to dissolve the remaining pectin components of the stem during the initial stage of water retting. Hence, the water-soluble fraction was found only in the nonretted stems (0.1% of the stem dry weight), and this fraction was absent in the total pectin content extracted from the midretted and retted fibre samples. Once the pectin-rich middle lamella is removed from around and between the fibre bundles, the bast fibres begin to separate from the core of the epidermis and one another.

The pectinolytic bacteria present in an aquatic environment are responsible for the breakdown of pectic materials and the subsequent release of fibres; as a result, the water retting procedure consistently yields excellent quality fibres in contrast to other retting techniques [24]. Previously, flax was also retted by enzyme mixtures comprising polygalacturonase from various fungal sources to investigate the effects on the qualities of the fibre. These findings demonstrated that retting, which causes pectin to break down around and between fibre bundles, can be caused only by the homogalacturonan areas of the flax stem wall, which are unmethylated or have a low degree of esterification [25]. Ruan et al. (2015) [9] defin

ed the term ‘degumming rate’ as the rate of change in the pectin content in flax stems during water retting. The only factor that contributed to the weight loss of the flax stems during water retting was the dissolution of pectin and other noncellulosic components. In our study, the amount of pectin in the jute stems changed significantly after 7 days of retting, accounting for approximately 78% of the total pectin degradation, and after 10 days of retting, the degumming rate exceeded 93%. The separation of the fibre bundles from the core was also significantly enhanced by the disintegration of pectin-rich tissues around the bast fibre bundles.

### Ultrastructural changes in jute stems during water retting

The gradual decomposition of pectin-rich parenchyma and the separation of fibre cells from the other bast tissues were observed via light microscopic examination of LS and TS sections of retted, mid-retted, and non-retted bast fibres and stems. Jute stems that were not retted showed intact structural arrangement. By releasing degradative enzymes into the environment over time, microbial colonization alters the structural integrity of bast fibre cells, causing the retting process to proceed. Morphological analyses using atomic force microscopy (AFM) of the flax cell walls during retting, revealed that the distance between fibres gradually increased with the disappearance of the middle lamellae, demonstrating the progressive action of enzymes on the digestion of the middle lamellae [26]. The progressive stages of microbial colonization and ultrastructural alterations of hemp stems during water retting were reported in a study by Fernando et al. (2019) [8]. Another study on the influence of chelating agents and mechanical pretreatment of flax fibre showed that during enzymatic retting, fibre bundles are separated from the core and cuticle and further dissociated into elementary fibres [27]. A study highlights the efficacy of a microbial food supplement in enhancing the growth of retting microbial populations, consequently resulting in improved bundle strength and finer fibrils, ultimately leading to higher fiber quality [28]. In our study, the preliminary stage of microbial water retting in jute stems lasted for nearly seven days. At this stage, microbial colonization occurred, and the fibre bundles began to separate from the phloem cells and the epidermal layer, while maintaining their structural integrity, with undamaged phloem present between the bundles. The middle lamellae connecting the fibres contain less methylated pectin and exhibit increased resistance to pectin-degrading enzymes, which could be the cause [29]. At the later stage of retting, the fibres finally separate. Fibre bundles become isolated from the remaining underlying tissues and from one another after 10 days of microbial water retting. The pectin-rich parenchyma is completely disintegrated by pectin-degrading enzymes produced by the bacterial consortia.

### Microbial colonization and bacterial-fungal interactions during retting

A recent study investigated the microbial dynamics during the retting process of jute using a metagenomic approach across three phases: pre-retting, aerobic retting, and anaerobic retting and identified dominant pectinolytic microflora in retting process [30]. The clear evidence provided in our study by the advanced microscopy images established the colonization of rod-shaped *Bacilli* and their interaction with retting water fungi. During the progression of retting, bacterial colonization on the inner and outer surfaces of the bark differed considerably. The first colonization point was when the outer surface was in direct contact with the retting water. At approximately day 3, rod-shaped *Bacilli* were visible on the surface, in contrast to the samples from day 0. Bacterial ingress to the inner bark takes some time, and SEM micrographs of mid-retted (day 5- day 7) bark samples revealed the establishment of microbial colonies on the inner surfaces and distorted exterior surfaces covered in microbial mats and fungal spores. Micrographs of the retted fibre samples showed almost complete dissolution of the pectineus matter. Figures 3 and 4 also show the presence of bacterial biofilms and fungal spores on the bark. This unequivocal evidence suggests that bacterial colonization begins on the outer surfaces, after which the bacteria enter the inner bark, peaking within 5–7 days, and by 10 days, complete removal of pectineus material occurs under ideal conditions. This allows the possibility of designing suitable techniques and quickly washing and recovering intact fibres and thus may save up to 5–7 days of retting time.

## Conclusions

Retting is a complex procedure with a notable influence on the ultimate quality of the jute fibre. Inadequate separation of fibers and weakening are consequences of both underretted and overretted fibers, respectively.Using a combination of microscopic and biochemical techniques we examined the structural and biochemical changes that occur during the water retting of jute fibres.

The study showed that the bacteria-mediated sequential breakdown of pectin around fibre bundles is the principal incident during water retting of bast fibres. It was observed that the pectin acts as a glue, binding the fibres together and preventing their separation. During the retting process, the pectin undergoes enzymatic degradation, leading to breakdown of the glue and separation of the fibres. The study also revealed that the retting process leads to a significant decrease in the pectin content in the jute stem. This change in composition may have important implications for the processing and properties of the fibres, and microbial breakdown of pectin is of paramount importance.

In conclusion, this study provides new insights into the retting process of bast fibres, shedding light on the structural and biochemical changes that occur during this critical step in the production of natural fibres. The findings of this study could be useful for improving the efficiency and quality of the retting process and for developing new techniques for the production of natural fibres.

## Data Availability

All the data generated or analyzed during this study are included within the article.
